# Primary surgical repair of tetralogy of fallot at the Uganda Heart Institute: a ten-year review of 30day mortality and morbidity

**DOI:** 10.1186/s12872-024-03991-z

**Published:** 2024-06-26

**Authors:** Rebecca Esther Khainza, Michael Oketcho, Twalib Aliku, Judith Namuyonga, Emma Ndagire, Tom Mwambu, Rwakaryebe Mbagga Muhoozi, Bernard Obongnyinge, Hilda Tumwebaze, Nestor Mbabazi, Teddy Akech, Aisha Nakato, Angelline Killen, Geoffrey Oburu Ofumbi, Peter Lwabi, John Omagino, Sulaiman Lubega

**Affiliations:** 1 Department of Paediatric Cardiology, Uganda Heart Institute (UHI), Kampala, Uganda; 2 Department of Cardiovascular and Thoracic Surgery UHI, Kampala, Uganda; 3 Department of paediatrics, Mulago National Referral Hospital, Kampala, Uganda; 4 Department of Paediatrics, Makerere University, Kampala, Uganda; 5 Uganda Christian University School of Medicine, Mukono, Uganda; 6 China -Uganda Friendship Hospital Naguru, Kampala, Uganda

**Keywords:** Tetralogy of Fallot, Early surgical outcomes, Post-operative ventilation time

## Abstract

**Background:**

Tetralogy of Fallot (TOF) is the most common form of cyanotic congenital heart disease (CHD) worldwide. It accounts for 7% of CHD cases in Uganda and leads to fatal outcomes in the long term without surgery. Surgery is often delayed in developing countries like Uganda due to limited resources.

**Objective:**

This study aimed to determine the early surgical outcomes of patients with TOF who underwent primary intracardiac repair at the Uganda Heart Institute (UHI) and to identify factors associated.

**Methodology:**

This retrospective chart review evaluated outcomes of primary TOF repair patients at UHI from February 2012 to October 2022. Patient outcomes were assessed from surgery until 30 days post-operation.

**Results:**

Out of the 104 patients who underwent primary TOF repair at UHI, records of 88 patients (84.6%) were available for review. Males accounted for 48.9% (*n* = 43). The median age at the time of operation was 4 years (with an interquartile range of 2.5-8.0 years), ranging from 9 months to 16 years. Genetic syndromes were present in 5/88 (5.7%). These included 2 patients with trisomy 21, 2 with Noonan’s, and 1 with 22q11.2 deletion syndrome. Early postoperative outcomes for patients included: residual ventricular septal defects in 35/88 (39.8%), right ventricular dysfunction in 33/88 (37.5%), residual pulmonary regurgitation in 27/88 (30.7%), residual right ventricular outflow tract obstruction in 27/88 (30.0%), pleural effusion in 24/88 (27.3%), arrhythmias in 24/88(27.3%), post-operative infections in 23/88(26.1%) and left ventricular systolic dysfunction in 9/88 (10.2%). Out of the children who underwent surgery after one year of age, 8% (7 children) died within the first 30 days. There was a correlation between mortality and post-operative ventilation time, cardiopulmonary bypass (CPB) time, aortic cross-clamp time, preoperative oxygen saturations, RV and LV dysfunction and the operating team.

**Conclusion:**

The most frequent outcomes after surgery were residual ventricular septal defects and right ventricular failure. In our study, the 30-day mortality rate following TOF repair was 8%. Deceased patients had lower pre-operative oxygen levels, longer CPB and cross-clamp times, longer post-operative ventilation, RV/LV dysfunction, and were more likely operated by the local team.

**Supplementary Information:**

The online version contains supplementary material available at 10.1186/s12872-024-03991-z.

## Introduction

### Background

Tetralogy of Fallot (TOF) is the most common form of cyanotic congenital heart disease (CHD) accounting for approximately 5–10% of all cases of CHD worldwide [1], and specifically 7% in Uganda [2]. The classic description of TOF involves four anatomical abnormalities: a ventricular septal defect (VSD), overriding aorta, infundibular stenosis, and right ventricular hypertrophy [3]. However, the clinical presentation and management strategy for TOF varies depending on the exact anatomy of the right ventricular outflow tract (RVOT) and concurrent cardiac lesions. Early intervention improves survival and physiological outcomes [4].

The optimal age for corrective surgery in children with TOF is unclear. It has been recommended that infants between 6 and 12 months of age undergo surgery to prevent complications associated with chronic hypoxia, including end-organ damage and right ventricular failure [5].

It is important to recognise that there remains a significant unmet need for congenital cardiac surgery in Africa, and diagnosis and subsequent management for TOF is often delayed beyond the first year of life. This delay has implications due to increased association with perioperative complications, such as right ventricular dysfunction, bleeding, low cardiac output syndrome, arrhythmias, and mortality, in children aged more than one-year undergoing surgical repair of TOF [6].

Data on outcomes following primary surgical repair of TOF in low-and middle-income countries (LMIC) remain scarce. Mortality post repair of TOF reported from high-income countries (HIC) appears to be much lower than the few data available from LMIC [7, 8]. Studies from both HIC and LMIC have identified multiple risk factors associated with increased post-operative mortality following surgical repair of TOF. Surgical factors include increased duration of aortic cross-clamp time, cardiopulmonary bypass (CPB) time, multiple CPB runs, and insertion of a transannular patch in children with less than 0.48m2 body surface area [5].

Patient factors include a small valve annulus size, narrow pulmonary arteries (PAs), and branch pulmonary [9]. Studies describing TOF repair in other African countries have reported frequent post-operative morbidities including prolonged pleural effusions, pericardial effusions, right ventricular failure, arrhythmias, and poorly controlled bleeding [8–10].

Access to congenital cardiac surgical services in Uganda is limited to a single tertiary centre, The Uganda Heart Institute. Cardiac surgeries have been performed since 2007, with approximately 60 closed-heart and 60 open-heart surgeries carried out annually for paediatric patients. The first primary surgical repair for TOF was performed in 2012. Initially, surgical services were closely supported by visiting surgeons from HIC, who have aided in the development of the local surgical team. The team consists of six cardiothoracic surgeons two of whom specialise in paediatric cases. Currently, most cases are performed by the resident surgical team, with intermittent assistance from visiting teams.

Gaining insight into patient characteristics and factors affecting early outcomes following primary intracardiac TOF repair will play an important role in guiding strategies to evolve cardiac services and ultimately improve outcomes in children undergoing cardiac surgery in Uganda. To date, no studies have been published to evaluate early surgical outcomes following primary surgical repair of TOF in Uganda.

The study aimed to provide comprehensive insights into clinical outcomes and potential risk factors influencing postoperative recovery in children undergoing surgical repair of TOF in Uganda.

We hypothesised that early morbidity and mortality after TOF repair are likely greater in Uganda compared to HIC due to resource constraints and delays in accessing heart surgery.

The primary outcome was 30-day in- and out-of-hospital mortality rates in patients aged less than 18 years who underwent primary intracardiac repair for TOF at Uganda Heart Institute.

Secondary endpoints included: (a) 30 days in hospital post-operative complications, assessed according to pre-established criteria, with a concurrent evaluation of associated risk factors; (b) ventilation time post-procedure, ICU, and hospital length of stay (LOS).

## Methods and materials

### Study design

This was a retrospective cohort study of consecutive patients aged less than 18 years who underwent primary surgical repair of tetralogy of Fallot at the Uganda Heart Institute between February 2012 to October 2022.

### Exclusion criteria

We excluded patients who needed palliative shunts such as modified Blalock-Thomas-Tausig shunts, ductal or right ventricular outflow tract stents, or right ventricle to pulmonary (RV-PA) conduits. Given limited resources, our institution adopts a highly conservative approach to systemic pulmonary shunts, and presently, there is no established single ventricle pathway.

#### Ethical approval

This study received approval from the Uganda National Council for Science and Technology, and ethical clearance was obtained from the Uganda Heart Institute Research and Ethics Committee.

### Data collection and variables

Perioperative data were collected from the patient’s records, incorporating clinical notes, echocardiogram (ECHO), electrocardiogram (ECG) and laboratory results.

Early surgical mortality was defined as death within 30 days of operation, both in and out of the hospital.

Early surgical morbidity was defined as a complication occurring within 30 days of the operation or before hospital discharge.

Intraoperative data included the surgical approach (trans-atrial and/or trans-ventricular) and techniques including (infundibular resection, commissurotomy, trans-annular patch repair, and augmentation of RVOT or branch pulmonary arteries), cardiopulmonary bypass time and aortic cross-clamp time.

Post-operative data comprised ventilation time post-procedure, ICU stay and hospital length of stay.

Data about ECHO findings were collected during both the pre-operative and post-operative periods. The timing for post-operative echocardiography varied based on the patient’s clinical condition and was conducted before discharge from the hospital. Out of the seven patients who passed away, one had echocardiogram findings recorded through transoesophageal ECHO as he died shortly after being transferred to the ICU. The remaining six patients had their screening transthoracic ECHOs done from the ICU. For the rest of the patients, we recorded the findings of the transthoracic ECHOs done before discharge, and the timing of the ECHO varied from patient to patient. ECHO parameters included right ventricular outflow tract obstruction (RVOTO), ventricular septal defects (VSDs), residual pulmonary regurgitation (PR), right ventricular function (RV) function, and left ventricular (LV) function.

RV function was assessed using either tricuspid annular systolic plane excursion (TAPSE) or a cardiologist’s subjective assessment.

Outcome variables comprised of in and out of hospital mortality, along with complications such as unscheduled cardiac re-operation (excluding re-operation for bleeding), bleeding necessitating re-operation, acute kidney injury (AKI) defined as requiring dialysis, infective complications, low cardiac output syndrome, new onset seizures, pericardial effusion after removal of the chest tube requiring tapping, prolonged drainage of pleural effusions, pneumothorax, cardiopulmonary bypass time, aortic cross-clamp time, ventilation time post-procedure, ICU stay and hospital length of stay.

Infective complications comprised of verified or assumed sepsis by the treating paediatricians, lower respiratory tract infections (LRTI) confirmed on clinical diagnosis or through chest X-rays (demonstrating pathological infiltrates, consolidation, or cavitation), as well as superficial or deep wound infection. Additionally, any other type of infection was defined as a confirmed diagnosis by the treating paediatrician.

### Sample size

Over ten years, starting from February 2012 and ending in October 2022, a total of 104 patients who were diagnosed with Tetralogy of Fallot (TOF) underwent operations at UHI. The reason for the selection of February 2012 as the starting point is that it was when the first intracardiac TOF repair surgery was conducted at UHI.

### Surgical technique used

The surgery to address tetralogy of Fallot was performed using a median sternotomy and standard cardiopulmonary bypass techniques. The patients were cooled to 32 °C and cooled further as needed. The repair of the tetralogy was carried out using a trans-atrial approach. In some cases, the muscle bundles were further resected through a right ventriculotomy and combined with augmentation of the right ventricular outflow tract. Glutaraldehyde-treated autologous pericardial patches were used for closure of the ventricular septal defect and augmentation of the right ventricular outflow tract, main pulmonary artery, and left pulmonary artery if required. In a few patients, the tetralogy repair was done through the trans ventricular approach with the assistance of visiting teams who used this as their standard practice. For some patients who were found to still have a significant gradient in the RVOT following a trans-atrial approach, a right ventriculotomy was done for further muscle bundle resection and augmentation of the RVOT.

Pulmonary annular z-score was used to predict transannular patch (TAP) need for borderline cases. TAP is inserted if the z-score is less than − 2 [11].

### Statistical analysis

Categorical variables are presented in numbers and percentages. To compare patient and surgical characteristics between children who died versus survived, Fisher’s exact test was employed for categorical variables. The normal distribution of continuous variables was assessed using Shapiro-Wilk testing and variables with a p-value < 0.05 was considered not normally distributed.

Nonparametric test Mann-Whitney U test was applied for non-normally distributed data. Univariate analysis was conducted to explore potential risk factors for postoperative mortality.

Data for non-normally distributed variables was described as median and interquartile ranges (IQR).

Statistical analyses were performed using STATA (V 13.0). A p-value of < 0.05 was considered statistically significant.

## Results

### Patient demographics

One hundred and four patients underwent primary surgical repair for TOF during the study period. Folders were available for 88/104 (84.6%) of patients.

Among the remaining 88 patients, the median age at surgery was 4 years IQR (2.5-8.0) years with ages ranging from 9 months to 16 years.

Genetic syndromes diagnosed based on phenotype only as genetic testing is not routinely available at our institution were present in 5/88(5.7%) of cases. These included two patients with trisomy 21, 2 patients with Noonan’s syndrome and 1 patient with 22q11.2 deletion syndrome.

**Fig. 1:**
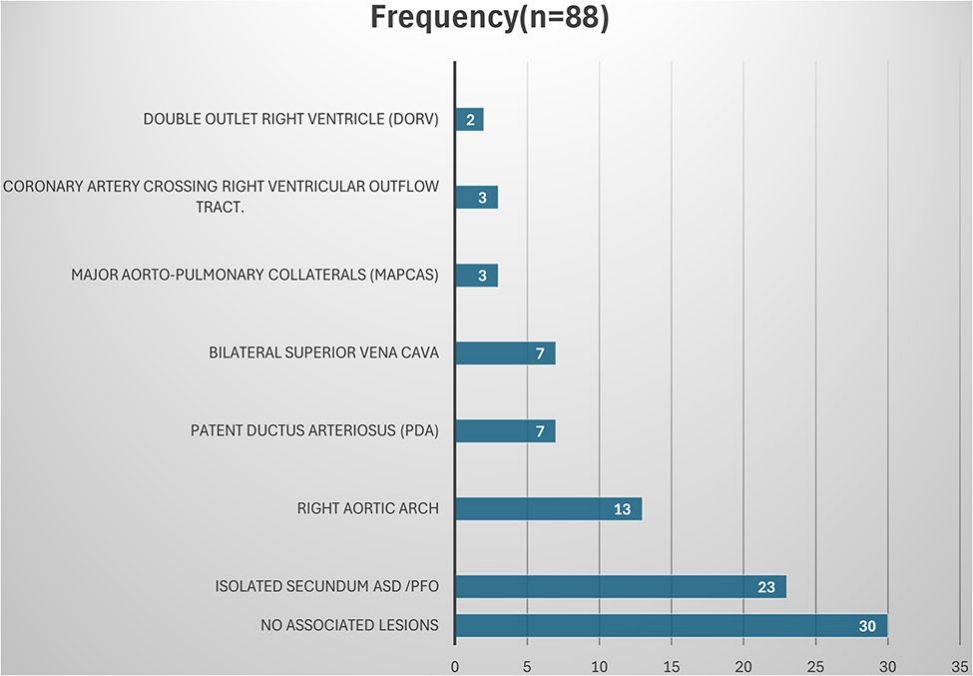
Echocardiographic findings of associated cardiac defects amongst patients who underwent primary TOF repair at UHI. *ASD = atrial septal defect, PFO = patent foramen oval*

The most common associated cardiac lesion was secundum ASD/PFO followed by right-sided aortic arch. Figure 1. The trans-atrial approach was the most frequently used surgical technique in tetralogy repair. Transannular patch repair was only performed in 8.0% of patients (*n* = 7).

Other procedures that were performed simultaneously included infundibular resection (95.5%(*n* = 84), augmentation of RVOT 77.3%(*n* = 68), commissurotomy 35.2% (*n* = 31), trans-annular patch repair 8% (*n* = 7), augmentation of main pulmonary artery 42%(*n* = 37) and augmentation of left pulmonary artery 3.4 (*n* = 3).

The most frequent preoperative echocardiogram finding was severe RVOTO, and the post-operative echocardiogram finding was right ventricular (RV) dysfunction. Most of the patients had no residual RVOTO, VSDs or PR and a small number had mild residual PR Table 1.

**Table 1:** Demographic and clinical factors

Variable	Frequency (%)	**30-day Mortality**		P – Value Fisher’s Exact test
	**N = 88**	Yes	No	
**Age(yrs.) Median /IQR**	N/A	3(2.3-4.0)	4.3(2.6-8.0)	0.157(M)
**Gender**				
Male Female	43(48.9) 45(51.1)	4(4.6) 3(3.4)	39(44.3) 42(47.7)	0.710
**Weight (Kg). Median/IQR**	N/A	11(9–14)	15(10.7–23)	0.065(M)
**Height (Cm). Median/IQR**	N/A	92(76–106)	102.5(90–127)	0.080(M)
**Body surface area(m** ^**2**^ **). Median/IQR**	N/A	0.53(0.42–0.65)	0.65(0.52–0.9)	0.0712(M)
**Phenotypic syndrome**				
Yes No	5(5.7) 83(94.3)	0(0) 7(8.0)	5(5.7) 76(86.3)	1.000
*Pre-operative haematocrit (%). Median/IQR	N/A	60.6(32.7–65.7)	47.25(39.9–57.9)	0.502(M)
**Pre-operative oxygen sats (%). Median/IQR**	N/A	65 (56–74)	86(79–94)	**0.001(M)**
**Surgical Approach**				
Trans Atrial only Trans Ventricular only Both Trans Atrial & Ventricular	61(69.3) 22(25) 5(5.7)	6(6.8) 0(0) 1(1.1)	55(62.5) 22(25) 4(4.6)	0.198
**Operating Surgical Team**				
Local Surgeons Combined international and local Surgeons	57(64.8) 31(35.2)	7(8.0) 0(0)	50(56.8) 31(35.2)	**0.049**
**Infection**				
Yes No	23(26.1) 65(73.9)	2(2.3) 21(23.8)	5(5.7) 60(68.2)	1.000
**Pulmonary valve Annulus z score**				
(>-2) (<-2)	80(90.9) 8(9.1)	7(8) 0(0)	73(82.9) 8(9.1)	1.000
**Pre-Operative RVOTO**				
Mild (Max PG < 36mmHg) Moderate (Max PG 36-64mmHg) Severe (Max PG > 64mmHg)	2(2.3) 6(6.8) 80(90.9)	0(0) 1(1.1) 6(6.8)	2(2.3) 5(5.7) 74(84.1)	0.500
**Post-operative Echo findings** Residual RVOTO				
None (Max PG < 25mmHg) Mild (Max PG < 36mmHg) Moderate (Max PG 36-64mmHg) Severe (Max PG > 64mmHg)	61(69.3) 15(17.1) 8(9.1) 4(4.5)	5(5.7) 1 (1.1) 1(1.1) 0 (0)	56(63.6) 14(15.9) 7(8) 4(4.5)	0.869
**Residual ventricular septal defects**				
None Small Moderate Large	53(60.2) 32(36.4) 2(2.2) 1(1.1)	5(5.6) 1(1.1) 1(1.1) 0(0)	48(54.5) 31(35.2) 1(1.1) 1(1.1)	0.166
**Pulmonary regurgitation**				
None Mild Moderate Severe	61 (69.3) 11(12.5) 7(7.95) 9(10.2)	5(5.7) 0(0) 0(0) 2(2.27)	56(63.6) 11(12.5) 7(7.95) 7(7.95))	0.162
**Right ventricular function**				
Normal Impaired	55(62.5) 33(37.5)	0(0) 7(7.95)	55(62.5) 26(29.5)	**0.001**
**Left ventricular function**				
Normal Impaired	79(89.3) 9(10.2)	2(2.27) 5(5.68)	77(87.5) 4(4.54)	**0.001**
Cardiopulmonary bypass time (min). Median/IQR	N/A	242(211–440)	155(118–191)	**0.002(M)**
Aortic cross-clamp time (min). Median /IQR	N/A	158(126–207)	97(72–125)	**0.002(M)**
ICU stay (days). Median /IQR	N/A	4(2–12)	5(3–6)	0.865(M)
Mechanical ventilation time (hrs). Median/IQR	N/A	52(48–228)	2(0–12)	**0.001(M)**
Overall hospital stays(days). Median /IQR	N/A	6(5–29)	16(11–26)	0.093(M)

**NB: X**_**2**_ **2-sided Fishers’ Exact Chi-square was utilized, and statistical significance was attained at P < 0.05**

**M = P-value (Mann-Whitney U test) clinical significance attained at p < 0.05**

**IQR = Interquartile range. ICU = Intensive care unit**

*=missing data.

**RVOTO = right ventricular outflow tract obstruction.** *RV function was assessed using either tricuspid annular plane systolic excursion (TAPSE) or a Cardiologist’s subjective assessment. No FAC (fractional area change) was done. Only 30/88 patients had TAPSE done.*

*LV function was assessed using ejection fraction and fractional shortening. RVOT assessment was maximum pressure gradients.*

*PR assessment was by maximum pressure gradients.*

*These findings were obtained from the most recent echocardiogram.*

*One patient had a large residual VSD due to the dehiscence of the VSD patch, this patient had a redo surgery about three months after the initial surgery.*

### Morbidity

Fifty-four of the 88 patients (61.3%) had a postoperative complication, with some patients 27/88 (31.7%) having more than one complication.

Pleural effusions and arrhythmias were the most common causes of morbidity followed by post-operative infections Table 2. The commonest arrhythmia was junctional ectopic tachycardia *n* = 10(11.4%) followed by heart blocks *n* = 6(6.8%). Other arrhythmias included ventricular fibrillation, paroxysmal ventricular tachycardias and paroxysmal supraventricular tachycardia as detailed in the supplementary. Some of the arrhythmias were self-limiting, others required antiarrhythmic drugs and a few required pacing.

**Table 2:** Morbidity of operated patients

Variable	Frequency(*N* = 88)	Proportion (%)
Pleural effusion (equal or > 7days)	24	27.3
Arrhythmias		
Atrial (PSVT)	24	27.3
Junctional (JET)	2	2.3
Ventricular	10	11.4
PVCs	2.0	2.3
Fibrillation	4.0	4.5
Heart block	6.0	6.8
Postoperative infection	23	26.1
Septicaemia	10	11.4
Pneumonia	7	8.0
Surgical wound sepsis	4	4.5
Malaria	1	1.1
Hand gangrene	1	1.1
Acute kidney injury	7	8.0
Low cardiac output syndrome	6	6.8
Cardiac arrest	6	6.8
Pneumothorax	4	4.5
Re-operation due to bleeding	1	1.1
New onset seizures	1	1.1
Chylothorax	1	1.1

*Pleural effusion ≥ 7 days is defined as prolonged*

*PSVT = Paroxysmal supraventricular tachycardia*

*JET = Junctional ectopic tachycardia*

*PVCs = Premature ventricular contractions*

Twelve patients 12(13.6%) with arrhythmias, required temporary pacing and none required permanent pacing.

Twenty-three of the 88 patients (26.1%) developed post-operative infections as detailed in the supplementary. Ten patients were treated for septicaemia, but cultures were only performed in 4 patients (2 enterococcus, 1 pseudomonas species and 1 negative culture).

Six patients suffered in-hospital cardiac arrests. Three patients recovered with no sequelae, 2 patients died, and 1 patient developed seizures which were effectively managed with phenobarbitone under the observation of a paediatric neurologist. Phenobarbitone was later weaned off and the patient was discharged from the neurology clinic seizure-free but had mild left-sided upper limb weakness for which he is still undergoing physiotherapy.

### Mortality

Thirty-day mortality was 8.0% (7/88) (Table 3). All patients who died were older than 1 year, but there was no significant difference in age between the group of patients who died and those who survived (Table 1). Among patients who died, 5 out of 7 (71.4%) had oxygen saturations less than 70% (ranging from 52 to 87%), and none required a transannular patch. The most frequent causes of death were RV dysfunction (*n* = 2) and RV dysfunction combined with acute kidney injury (*n* = 5).

**Table 3:** Description of TOF patients who died following surgery

Characteristic	Patient 1	Patient 2	Patient 3	Patient 4	Patient 5	Patient 6	Patient 7
Age (years)	7.5	4	3.3	3	1.8	3	2.3
Gender	Male	Male	Female	Male	Male	Female	Female
Phenotypic syndrome	None	None	None	None	None	None	None
Preoperative oxygen %	56	74	52	57	87	65	65
Body surface area (m^2^)	0.69	0.65	0.53	0.50	0.39	0.58	0.42
Weight (Kg)	15	14	11	11	8.5	13	9
Preoperative HCT	27	32.7	57.7	65.7	62.5	67.8	60.6
PV annulus z score	+ 0.39	-1.41	+ 0.48	+ 0.20	+ 0.75	-0.41	-0.17
Pre-operative RVOTO	Severe	Severe	Severe	Severe	Severe	Severe	Severe
Associated lesions	PFO, dextrocardia	PDA	None	None	None	PFO	RAA, PFO
Surgical approach	TA	TA	TA	TA	TA/TV	TA	TA
Trans annular patch	None	None	None	None	None	None	None
Commissurotomy	None	Yes	Yes	Yes	None	Yes	None
Cardiopulmonary bypass time (Min)	253	194	585	238	440	242	211
Aortic cross-clamp time (min)	207	118	192	158	239	132	126
Post-operative mechanical ventilation time (hrs.)	360	48	52	216	0.17(10 min)	288	48
Timing of death in days. (< or > 24 h.)	14 days (> 24 h)	4 days (> 24 h)	2 days (> 24 h)	27 days (> 24 h)	10 min (< 24 h)	12 days (> 24 h)	3days (> 24 h)
ICU stay (days)	14	4	2	12	10 min	8	3
Ionotropic support duration	11	3	2	9	10 min	8	3
AKI	Yes	No	Yes	No	Yes	Yes	Yes
RV dysfunction	Yes	Yes	Yes	Yes	Yes	Yes	Yes
LV dysfunction	No	Yes	Yes	No	Yes	Yes	Yes
Arrhythmia (JET)	No	Yes	No	No	No	Yes	No
Cardiac arrest	No	Yes	No	No	Yes	No	No
Complete heart block	No	No	No	No	No	No	Yes
Severe/Free PR	No	Yes	No	Yes	No	No	No
Infections(septicaemia)	No	No	No	Yes	No	Yes	No
Residual VSDs	Moderate	Small	No	No	No	No	No
Likely causes of death	AKI, RV dysfunction	RV/LV dysfunction, JET, cardiac arrest	AKI, RV&LV dysfunction	RV dysfunction, septicaemia	AKI, RV/LV dysfunction, cardiac arrest	AKI, RV/LV dysfunction, JET, septicaemia	AKI, RV/LV dysfunction.

***HCT = Haematocrit. RVOTO = Right ventricular outflow tract obstruction. ICU = Intensive care unit. AKI = acute kidney injury. JET = Junctional ectopic tachycardia. PR = pulmonary regurgitation. RV = right ventricular. LV = Left ventricular. VSD = ventricular septal defects. Body surface area. TA = trans atrial. TV = Trans ventricular. RAA = Right aortic arch. PDA = Patent ductus arteriosus. PFO = patent foramen ovale***

***Note***: *High HCT values (> 53.7%), Low HCT values (< 35%), Low oxygen Values (< 92%), Prolonged cardiopulmonary bypass time CPB (≥ 180), Prolonged aortic cross-clamp time ≥ 90. Prolonged ICU stay ≥ 7 days. Prolonged ventilation time post procedure ≥ 24 h. Severe RVOTO max PG > 64mmHg*

Preoperative haematocrit was higher and the duration of CPB and aortic cross clamp was longer in patients who died. The patients who died also had a longer duration of postoperative ventilation.

Two patients died who may have benefited from extracorporeal membrane oxygenation (ECMO), which is not currently available in our setting. The first patient, with severe preoperative RVOT, a baseline oxygen saturation of 52%, and a haematocrit of 57.5, presented for surgery at 3.3 years of age. This patient underwent four attempts to wean off CPB, all of which repeatedly failed due to severe RV dysfunction. The oxygenator was changed, and the child remained on CPB for an additional 4 h to rest the RV. He was eventually weaned off on the fifth attempt but required high inotropic support (milrinone, adrenaline, and noradrenaline). The cumulative CPB time was 585 min (9.8 h). Subsequently, in the ICU, the child experienced worsening RV dysfunction, and LV dysfunction, developed acute kidney injury (AKI) (progressing from oliguria to anuria) and died 48 h later.

The second patient who may have benefited from ECMO presented for surgery at 1.8 years with severe RVOTO, a baseline oxygen saturation of 87%, and a haematocrit of 62.5. This patient’s RV pressures persistently remained high on the table despite adequate resection of the muscle bundles. This necessitated going back on the bypass machine twice thus the prolonged cumulative cardiopulmonary bypass time of 440 min. A trans oesophageal echocardiography at the end of surgery showed no residual RVOT stenosis but with RV pressures of 58 mmHg and RVOT pressure of 22/11 mmHg. Direct pressure measurements for RV pressures were 40mmHg and aorta 60mmHg. He was weaned off the pump, his sternum left open, and his skin closed with a membrane. He was put on high inotropic support. The patient had both severe RV and LV dysfunction and features of AKI and died within 10 min in the ICU.

## Discussion

### Summary of principal findings

The principal finding of this study was that 30-day mortality following primary surgical repair of TOF at our institution during the 10 years was 8%.

The patients who died had a longer duration of cardiopulmonary bypass time, aortic cross-clamp time, ventilation time post-procedure, low preoperative oxygen saturations, RV dysfunction, and LV dysfunction and were more likely to have been operated by the local team alone.

### Mortality incidence between high and LICs

Data on outcomes following TOF repair in LMICs remain scarce with the total number of included patients being few compared to HICs. A systematic review and meta-analysis on outcomes after surgical repair of TOF reported an overall 30-day mortality of 4.2% in Africa and the Middle East combined, but only included data from Egypt and Ivory Coast [12]. The few other studies from Africa reporting on perioperative mortality following TOF repair report much higher mortality ranging from 9.0 to 12.9% [8–10]. The overall reported mortality in our patient cohort appears to be slightly lower, but it is significantly higher than data from HICs. Some of the studies from HICs reported no mortality in their cohorts [7, 13].

### Differences in patient characteristics between LMICs and HICs

The patient demographics differ between LICs and HICs. Patients from LICs tend to be older with lower oxygen saturation and higher haematocrit. This is important to consider because of the impact of chronic cyanosis as a known risk factor for poor postoperative outcomes.

A study from China, an upper middle-income country, observed that lower pre-operative saturations (median 82%, IQR 72–88 versus median 89%, IQR 81–95) were associated with poor outcomes [14]. In the Ethiopian study by Tefera et al., the median pre-operative saturation of patients who died was 76%, compared to 86% in survivors; however, the difference was not statistically significant [9]. These saturations are still much higher than the preoperative saturations in this study cohort. In another study conducted in Cameroon, a lower-middle-income country, the mean oxygen saturation before surgery was as low as 67 ± 5% [8].

In low-middle-income countries, there are few modified Blalock-Taussig shunts (MBTS) recommended to improve saturations, and when done, they tend to perform poorly.

Patients with chronic hypoxia are at increased risk of arrhythmias and other complications due to polycythaemia, such as strokes.

Patients with TOF operated from LMICs tend to be older than those from HIC. Most of the LMICs operate on TOF patients after one 1year of age [8–10]. Older age at surgery has been linked to higher mortality due to chronic hypoxia and RV failure [5].

### Factors associated with mortality

Our study links mortality to several factors, including time on cardiopulmonary bypass, aortic cross-clamp duration, mechanical ventilation time, low pre-op oxygen saturation, RV and LV dysfunction, and the operating team.Due to delayed surgeries, these children may require significant muscle bundle resection. This can result in extended periods of being on cardiopulmonary and aortic cross-clamp, making them more susceptible to health complications.

Late surgeries can increase the risk of RV failure due to chronic cyanosis. In our study, all patients who passed away experienced RV failure, and in two cases, patients had to be kept on the pump to support the RV due to lack of access to ECMO. These issues contributed to longer cardiopulmonary time.

Patients who died were sicker, required more ventilatory support, and had prolonged ventilation time. Our findings are comparable with studies in LMICs. In the Ethiopian study, prolonged cardiopulmonary bypass time/aortic cross-clamp time and pulmonary valve annulus diameter less than three standard deviations (SD) were independently associated with perioperative mortality [9]. A study by Amirghofran et al. in Shiraz, Iran found a correlation between mortality with pump and ventilation time post-procedure [15]. In Berne Switzerland, found age, cardiopulmonary bypass time, aortic cross-clamp time and higher maximum post-operative troponin levels [16].

One of the common denominators among patients who died in our study was acute kidney injury (AKI) requiring dialysis. This could be attributed to limited access to haemodialysis at UHI, with only peritoneal dialysis available at that time. AKI can also be linked to RV failure. Factors may be due to delayed surgery, but age at surgery was not directly linked to mortality probably due to the small sample size.

### Patient selection

The provision of paediatric cardiac surgical services in LMICs has historically been limited to short-term surgical visits from HICs and published data on outcomes from these initiatives are also limited. While the local team operated on all the patients who died in our study, this is unsurprising given the deliberate strategy of selecting patients who are expected to have favourable outcomes to be operated on by the visiting team for teaching /training, while the local team tends to operate sicker patients in comparison.

Patient selection plays a vital role in determining operative outcomes for paediatric cardiac surgery in LMICs like Uganda. With limited resources, prioritizing patients who are likely to benefit most, such as those with favourable anatomy is crucial. This likely influenced more favourable outcomes than if we were operating on all patients, including those with more complicated anatomy. The fact that our patients are older, with a median age of 4 years, suggests that their anatomy was not as complex, enabling them to reach this age. However, older age is associated with more complications, particularly RV dysfunction, following surgery. In our setting, ECHMO is not available to support those patients who may require it.

It has been noted that in low-income countries, TOF surgery is performed on older children as compared to high-income countries. In a study from Ethiopia by Tefera et al., the median age was 7 years with an age range of 1–22 years, while in a study from Cameroon by Tchoumi et al., the mean age was 9.18 +/- 6.5 years with a range of 13.5 months to 26 years [8, 9]. This was not different from this cohort.

In some surgical procedures for TOF repair, patients may experience medium to long-term outcomes. For example, TAP repair relieves RVOTO but may lead to severe pulmonary regurgitation in the long run which may require re-operation. The Finnish research database looked at 600 patients who underwent TOF repair before 15 years of age and in their findings TAP repair carried a high risk of re-operation but had no impact on late survival [17]. Patients with adequate pulmonary valve annulus i.e. PV annular z-sore >-2 are usually preferred during patient selection to avoid the need for a TAP. In our study TAP repairs were few because these children were older with favourable anatomy i.e. many had good pulmonary valve annulus, MPA and branch PAs.

In comparison, a study by Tefera et al. at the children’s Heart fund cardiac centre run in collaboration with international charities and philanthropists, reported that 22/57, 38.6% of patients had a trans annular patch and 2 patients had RV-PA homografts inserted [9].

Similar to our context, this program was initially dependent on international surgeons, but now local teams operate. Data from small cohort of patients undergoing surgical repair in Cameroon, also reported a high incidence of repair with TAP (12/22, 54.5%). However, these surgeries were supported by international surgeons [8]. Patients with adequate pulmonary valve annulus i.e. PV annular z-score >-2 are usually preferred during patient selection at our institution to avoid the need for TAP. In our study, TAP repairs were few because these children were older with favourable anatomy i.e. many had good pulmonary valve annulus, MPA, and branch PAs.

### Morbidity

The most frequent causes of morbidity in our study were pleural effusions, arrhythmias, and postoperative infections. This was comparable with what was found in other LMICs in Africa.

In a study conducted in Nigeria by Olukemi et al., pleural effusion was found to be the leading cause of morbidity, followed by pericardial effusions and cardiac dysfunction [10]. In the study conducted in Cameroon, only a small number of patients (22) were involved, and the acute post-surgical complications that were recorded included pericardial effusion and pleural effusion in 4 and 3 patients respectively [8]. It’s possible that the low incidence of complications was due to the small sample size.

Arrhythmias are common in TOF patients, typically within 24 h [18]. In most of our patients, we were able to successfully correct the rhythm disturbances.

The causes of morbidity in HIC are quite like those found in the LMICs. A study conducted by Sameh Ismail et al. at King Saudi University also identified pleural effusions and arrhythmias as the primary causes of morbidity [13].

### Suggestions for improvement

Improving access to cardiac surgery can help reduce wait times and enable children to undergo surgery at an earlier stage. Early diagnosis plays a critical role in identifying patients who require surgery. To achieve this, hospitals should encourage routine screening of newborns for oxygen saturation levels after birth, a practice that is not currently widespread.

The success of any surgical team depends on the expertise and skills of its members. To ensure exceptional performance, it is crucial to train more personnel, including cardiologists and cardiothoracic surgeons. These additional team members will not only enhance the surgical team’s capabilities but also improve the quality of care provided to patients. Investing in training now will pay dividends in the future, resulting in better patient outcomes and a more efficient healthcare system overall.

It is imperative to increase the number of surgeries performed. Doing so will significantly improve system efficiency, leading to a considerable reduction in operating time, including CPB and aortic cross-clamp times. Moreover, surgeries must be made accessible to both smaller babies and those with complex anatomy without any delay.

ECMO is highly recommended in our setting to reduce mortality due to RV failure. However, it requires personnel training and sustainable costs.

### Study strengths and limitations

This study provides the first documented report of early surgical outcomes of tetralogy of Fallot patients following primary intracardiac repair in Uganda.

The reported mortality rate cannot be considered entirely accurate due to the absence of data for 16 patients.

The fact that this was a single-centre study/ only centre where open-heart surgery is done in the country limits the generalizability of our results.

There was bias during patient selection, only those who qualified for primary intracardiac repair were taken.

Echocardiography timing varied based on the patient’s condition. However, at least one echocardiogram was done before discharge, suggesting no consideration for postoperative remodelling.

Some key statistics could not be measured given the retrospective design.

Medium- and long-term outcomes were not included in this study. A larger sample size was needed to be able to describe rare outcomes.

## Conclusion

This study provides the first report on the postoperative outcomes of tetralogy of Fallot patients who underwent primary intracardiac repair in Uganda. The study found that 8% of patients died within 30 days of the repair. Mortality was associated with various factors such as cardiopulmonary bypass time, aortic cross-clamp time, mechanical ventilation time after the procedure, low pre-operative oxygen saturations, RV dysfunction, LV dysfunction and operating team.

Patients who undergo surgery at a later stage are at a higher risk of early postoperative complications, including mortality.

Further studies are required to understand the medium- and long-term outcomes of patients who have undergone TOF surgery.

### Electronic supplementary material

Below is the link to the electronic supplementary material.

Supplementary Material 1

Supplementary Material 2

## Data Availability

Data will be available upon reasonable request from corresponding author.

## Abbreviations

TOF: Tetralogy of Fallot
CHD: Congenital heart disease
UHI: Uganda Heart Institute
VSDs: Ventricular septal defects
RV: Right ventricular
LV: Left ventricular
RVOT: Right ventricular outflow tract
RVOTO: Right ventricular outflow tract obstruction
LMIC: Low- and middle-income countries
HIC: High-income countries
ICU: Intensive Care Unit
HDU: High dependence Unit
LOS: Hospital length of stay
ECHO: Echocardiogram
ECG: Electrocardiogram
PR: Pulmonary regurgitation
AKI: Acute Kidney Injury
TAP: Trans annular patch
CPB: Cardiopulmonary bypass
ECMO: Extracorporeal membrane oxygenation
ASD: Atrial septal defect
