# Supplementary material for: Primary surgical repair of tetralogy of fallot at the Uganda Heart Institute: a ten-year review of 30day mortality and morbidity
Source: BMC Cardiovasc Disord. 2024 Jun 26;24:322. doi: 10.1186/s12872-024-03991-z (PMC11202334; doi:10.1186/s12872-024-03991-z)
Supplement: Supplementary file 1 — Supplementary Material 1 [file 12872_2024_3991_MOESM1_ESM.docx]

**Table 1. Supplementary**

**Nature infections among TOF patients during the postoperative period.**

| Nature of infection | Number N=88(%) | Method of diagnosis. | Laboratory Findings |
| --- | --- | --- | --- |
| Septicaemia | 1. (11.4) | 4/10 (Clinical + BC)  6/10 (Clinical +CBC) | 2 (enterococcus)  1 (pseudomonas)  1(culture negative)  Elevated WBC with neutrophilia. |
| Pneumonia | 7 (8.0) | 6/7(Clinical)  1/7 (clinical +CXR) | Heterogenous opacities involving right lung parenchyma. |
| Surgical wound Sepsis | 4 (4.5) | Clinical | - |
| Malaria | 1 (1.1) | Clinical + Microscopy | Plasmodium falciparum positive. |
| Hand gangrene | 1 (1.1) | Clinical | - |

*BC=Blood culture. CBC=Complete blood counts. WBC= White blood cell counts*

*CXR =Chest Xray.*

*Pneumonia was diagnosed mainly clinically by the attending paediatricians with a few cases having chest x-rays done. Pneumonia was defined as cough, fever, difficulty in breathing, and crepitations on auscultation. Diagnosis of septicaemia was mainly based on complete blood counts together with clinicians’ assessment. Septicaemia was diagnosed clinically for patients who had fevers and elevated neutrophils on CBC. Blood cultures were done for a few cases due to cost implications. Malaria was a blood smear under microscopy which showed plasmodium falciparum****.*** *Wound sepsis was defined as signs of infection like pus from the wound without systemic involvement.*
