# Supplementary material for: Primary surgical repair of tetralogy of fallot at the Uganda Heart Institute: a ten-year review of 30day mortality and morbidity
Source: BMC Cardiovasc Disord. 2024 Jun 26;24:322. doi: 10.1186/s12872-024-03991-z (PMC11202334; doi:10.1186/s12872-024-03991-z)
Supplement: Supplementary file 2 — Supplementary Material 2 [file 12872_2024_3991_MOESM2_ESM.docx]

Table 2: Supplementary

**Incidence of arrhythmias in operated TOF patients**

| Variable | Frequency (N=88) | Proportion (%) |
| --- | --- | --- |
| Atrial arrhythmias  PSVT | 2 | 2.3 |
| Junctional arrhythmias (JET) | 10 | 11.4 |
| Ventricular arrhythmias  PVCs  V. Fib | 2  4 | 2.3  4.5 |
| Heart block | 6 | 6.8 |

***PSVT****=Paroxysmal supraventricular tachycardia*

***JET****= junctional ectopic tachycardia*

***PVCs****=paroxysmal ventricular tachycardia*

***V. Fib*** *=ventricular fibrillation*
